# Circ_0044235 regulates the development of osteoarthritis by the modulation of miR-375/PIK3R3 axis

**DOI:** 10.1186/s13018-024-04694-z

**Published:** 2024-04-15

**Authors:** Wenjie Qian, Kai Mei, Lei Zhu, Ying Chu, Jinpeng Lv, Changjun Yun

**Affiliations:** 1 Department of Joint Orthopedics, Wujin Hospital Affiliated with Jiangsu University, Changzhou City, Jiangsu 213002 China; 2 Department of Joint Orthopedics, the Wujin Clinical College of Xuzhou Medical University, Changzhou City, Jiangsu 213002 China; 3 Department of science & education, Wujin Hospital Affiliated with Jiangsu University, Changzhou City, Jiangsu 213002 China; 4 Department of science & education, the Wujin Clinical College of Xuzhou Medical University, Changzhou City, Jiangsu 213002 China; 5 Changzhou University, Changzhou City, Jiangsu 213164 China

**Keywords:** Osteoarthritis, circ_0044235, miR-375, PIK3R3

## Abstract

**Background:**

Circular RNAs (circRNAs) play an important role in osteoarthritis (OA). However, the role of circRNA in OA is still unclear. Here, we explored the role and mechanism of circ_0044235 in OA.

**Methods:**

CHON-001 cells were treated with IL-1β to establish OA model in vitro. The levels of circ_0044235, miR-375 and phosphoinositide 3-kinase (PI3K) regulatory subunit 3 (PIK3R3) were detected by quantitative real-time PCR. Cell count kit-8 assay and flow cytometry assay were used to detect cell viability and apoptosis. The concentrations of inflammation factors were determined by enzyme-linked immunosorbent assay. Western blot was used to detect protein levels. The interaction between miR-375 and circ_0044235 or PIK3R3 was analyzed by dual-luciferase reporter assay and RNA immunoprecipitation assay.

**Results:**

Circ_0044235 was significantly decreased in OA cartilage tissue and IL-1β-treated CHON-001 cells. Overexpression of circ_0044235 promoted IL-1β-stimulated CHON-001 cell viability and inhibited apoptosis, inflammation, and extracellular matrix (ECM) degradation. In mechanism analysis, circ_0044235 could act as a sponge for miR-375 and positively regulate PIK3R3 expression. In addition, miR-375 ameliorated the effect of circ_0044235 overexpression on IL-1β-mediated CHON-001 cells injury. In addition, miR-375 inhibition mitigated IL-1β-induced CHON-001 cell injury, while PIK3R3 silencing restored the effect.

**Conclusion:**

Circ_0044235 knockdown alleviated IL-1β-induced chondrocytes injury by regulating miR-375/PIK3R3 axis, confirming that circ_0044235 might be a potential target for OA treatment.

## Introduction

Osteoarthritis (OA) is one of the most common musculoskeletal diseases, it is mainly caused by degenerative changes in the bone and joint, which can lead to deterioration of joint function and a decrease in the patient’s quality of life [1, 2]. The disease has a long and slow course, with mild joint distension at the early stage and mild symptoms, which are often ignored by patients, with the aggravation of the course of the disease, the pain will worsen, and joint activity will be limited, inflammation will increase, and adhesion will occur [3].

Circular RNA (circRNA) is a kind of non-coding RNA (ncRNA) with a circular structure, which is formed by back-splicing and is involved in transcription and post-transcriptional regulation [4]. Moreover, due to the lack of 5’ cap structure and 3’ poly-tail structure, circRNA has high stability and is not easily degraded by RNA exonuclease [5]. Many studies have shown that the levels of circRNAs are different in OA cartilage, and some circRNAs are also participated in various pathological processes of OA, such as extracellular matrix (ECM) degradation, inflammation, and apoptosis [6–8]. It was found that circ_0044235 could be used as a novel biomarker for systemic lupus erythematosus and rheumatoid arthritis [9, 10]. Besides, circ_0044235 was confirmed to be downregulated in rheumatoid arthritis patients, and its overexpression alleviated joint inflammation, cell apoptosis, and joint damage [11]. Therefore, circ_0044235 may be a regulator for musculoskeletal-related diseases. Here, we found that circ_0044235 had decreased expression in OA patients. However, the role and underlying molecular mechanisms of circ_0044235 in OA progression have not been investigated.

One of the mechanisms of circRNA is that it can act as a sponge for microRNA (miRNA) to mediate the regulation of downstream genes [12, 13]. MiRNAs are a sort of single-stranded ncRNAs, which can participate in regulating biological behaviors through the expression of targeted genes [14, 15]. Studies have shown that miRNAs are taken part in the development of musculoskeletal-related diseases [15, 16]. It had been reported that miR-375 mediated chondrocyte metabolism and oxidative stress in a mouse model of OA [17]. Moreover, miR-375 was up-regulated in IL-1β-induced chondrocyte injury [18]. Therefore, miR-375 might be an important miRNA regulating OA progression. Phosphoinositide 3-kinase (PI3K) regulatory subunit 3 (PIK3R3) is an inhibitor of PI3K [19] in the PI3K/AKT pathway, and is intimately related to OA pathogenesis [20, 21]. PIK3R3 was confirmed to be downregulated in OA knee cartilage and participated in OA progression [22, 23]. Here, we showed that circ_0044235 could bind to miR-375, and miR-375 could target PIK3R3. However, whether circ_0044235 mediates OA progression by regulating miR-375/PIK3R3 axis remains unclear.

Hence, our study was designed to study the role and mechanism of circ_0044235 on IL-1β-induced chondrocytes injury. Basing on the above, we proposed and verified the hypothesis that the circ_0044235 inhibited OA progression through the miR-375/PIK3R3 axis.

## Materials and methods

### Source of organization

The articular cartilage tissues of OA patients (*n* = 22) (8 males and 14 females, age range 65–77 years) in Wujin Hospital Affiliated with Jiangsu University were selected, and the normal tissues were taken from the normal articular cartilage of amputees (*n* = 22) (10 males and 12 females, age range 61–75 years) due to accidental injuries. All patients gave informed consent. And this experiment was supported by the Ethics Committee of Wujin Hospital Affiliated with Jiangsu University.

### Cell culture and transfection

Human chondrocytes (CHON-001) were offered by Nanjing Kebai Biotechnology Co., Ltd. (Nanjing, China). Cells were cultured in DMEM medium (Gibco, Carlsbad, CA, USA) containing 10% FBS (Gibco) at 37℃ with 5% CO_2_. In this, 10 ng/mL IL-1β was used to stimulate CHON-001 cells for 24 h to construct OA cell models [24, 25].

Lipofectamine 3000 (Invitrogen, Carlsbad, CA, USA) was used for cell transfection. Circ_0044235 overexpression vector (oe-circ_0044235), miR-375 mimic or inhibitor (anti-miR-375), small interfering RNA against PIK3R3 (si-PIK3R3) and their controls were obtained from Geneseed (Guangzhou, China).

### Quantitative real-time PCR (qRT-PCR)

The total RNA was extracted using TRIzol reagent (Invitrogen) and reversely transcribed into cDNA using cDNA synthesis Kit (Takara, Dalian, China). QRT-PCR was performed using SYBR Green (Takara). Relative expression was calculated by 2^−ΔΔCt^ method with GAPDH or U6 as internal reference. Primers were shown in Table 1.

**Table 1:** Primer sequences used for qRT-PCR

Name		Primers for PCR	Product size
hsa_circ_0044235	Forward	TGAGTTTGGTGATTCAGCTTGC	152 bp
	Reverse	AACAAGGCTTCTTCTGAGTGT	
CDC27	Forward	CAGTCTGTTGCCAGAATCGG	166 bp
	Reverse	GTGCGTTTGGGGGAGATGTA	
miR-375	Forward	GCGTTTGTTCGTTCGGCTC	66 bp
	Reverse	AGTGCAGGGTCCGAGGTATT	
miR-1200	Forward	GCGCTCCTGAGCCATTCTG	66 bp
	Reverse	AGTGCAGGGTCCGAGGTATT	
miR-335	Forward	CGCGTCAAGAGCAATAACGAA	67 bp
	Reverse	AGTGCAGGGTCCGAGGTATT	
miR-338-5p	Forward	CGCGAACAATATCCTGGTGC	66 bp
	Reverse	AGTGCAGGGTCCGAGGTATT	
miR-498	Forward	TTCAAGCCAGGGGGCG	67 bp
	Reverse	AGTGCAGGGTCCGAGGTATT	
miR-507	Forward	CGCGTTTTGCACCTTTTGG	65 bp
	Reverse	AGTGCAGGGTCCGAGGTATT	
miR-557	Forward	TTTGCACGGGTGGGCC	67 bp
	Reverse	AGTGCAGGGTCCGAGGTATT	
miR-574-5p	Forward	CGCGTGAGTGTGTGTGTGTGA	67 bp
	Reverse	AGTGCAGGGTCCGAGGTATT	
miR-892a	Forward	CGCGCACTGTGTCCTTTCT	65 bp
	Reverse	AGTGCAGGGTCCGAGGTATT	
PIK3R3	Forward	CGGTCGGGTTGGTTCTTACA	165 bp
	Reverse	CTGGTCTGCAGAGAGCGAAT	
GAPDH	Forward	GACAGTCAGCCGCATCTTCT	104 bp
	Reverse	GCGCCCAATACGACCAAATC	
U6	Forward	CTCGCTTCGGCAGCACA	94 bp
	Reverse	AACGCTTCACGAATTTGCGT	

### Actinomycin D (ActD) and RNase R assays

ActD (2 mg/mL; AAT Bioquest, Sunnyvale, CA, USA) was used to treat CHON-001 cells for indicated times. Then, the RNA was isolated, and qRT-PCR was performed to measure circ_0044235 expression and linear CDC27 mRNA expression.

3 U/µg RNase R (Geneseed) was used to dispose RNA for 30 min. Then, qRT-PCR was used to detect circ_0044235 expression and linear CDC27 mRNA expression.

### Cell counting kit-8 (CCK-8) assay

After transfection and treatment for 48 h, the cells were trained with 10 µL CCK-8 solution for 4 h according to the instructions of CCK-8 Kit (Solarbio, Beijing, China). The absorbance at 450 nm was monitored by the microplate reader.

### Flow cytometry

After transfection 48 h, CHON-001 chondrocytes were digested by trypsin. The chondrocytes were stained by Annexin V-FITC and PI staining solution (Beyotime, Shanghai, China) for 15 min. Flow cytometry was used to measure the chondrocyte apoptosis rate.

### Western blot

The total protein was extracted by RIPA lysis buffer (Beyotime), and then resolved by 10% SDS-PAGE and transferred to PVDF membranes. The membrane was sealed with 5% skim milk powder solution. The membrane was incubated with primary antibody against Bax (1:1,000, ab32503, Abcam, Cambridge, CA, USA), Bcl-2 (1:1,000, ab32124, Abcam), cleaved-caspase-3 (1:1,000, ab2302, Abcam), MMP-13 (1:3,000, ab39012, Abcam), Collagen II (1:1,000, ab34712, Abcam), Aggrecan (1:1,000, ab36861, Abcam), PIK3R3 (1:1,000, H00008503-A01, Abnova, Taiwan, China), or GAPDH (1:2,500, ab9485, Abcam) and cultivated at 4℃ overnight. Then, the membrane was fostered with Goat anti-rabbit or mouse IgG (1:50,000, ab205718 or ab205719, Abcam) at room temperature for 90 min, and ECL luminescent solution (Beyotime) was added for development. The relative protein expression level was calculated using GAPDH as internal reference.

### Enzyme-linked immunosorbent assay (ELISA)

According to the manufacturer’s instructions, TNF-α and IL-6 in supernatant were detected by human TNF-α and IL-6 Elisa kits (Hangzhou Lianke Biotechnology Co., Ltd., Hangzhou, China).

### Dual-luciferase reporter assay

The wild-type or mutant-type vectors of circ_0044235 or PIK3R3 (circ_0044235 WT/MUT or PIK3R3 3’UTR WT/MUT) were constructed using psiCHECK2 reporter vector. The above vector was co-transfected with miR-NC and miR-375 mimic into CHON-001 chondrocytes, and luciferase activity was estimated according to the instructions of Dual-Luciferase Reporter Gene Assay Kit (Beyotime).

### RNA immunoprecipitation (RIP) assay

RIP kit (Millipore, Billerica, MA, USA) was used to examine the binding of circ_0044235 or PIK3R3 to Ago2 protein. CHON-001 cells were collected and lysed, cell extract was incubated with antibody for co-precipitation with magnetic beads-coupled Ago2 antibody or IgG antibody (Millipore). After RNA was extracted, qRT-PCR was used for the detection of circ_0044235, miR-375 and PIK3R3 expression.

### Statistical analysis

These results were expressed as the mean ± SD. GraphPad Prism 8.0 was used for statistical analysis, and differences were analyzed by student’s *t*-test or analysis of variance. *P* < 0.05 was a significant difference.

## Results

### Circ_0044235 expression was diminished in OA patients

**Fig. 1:**
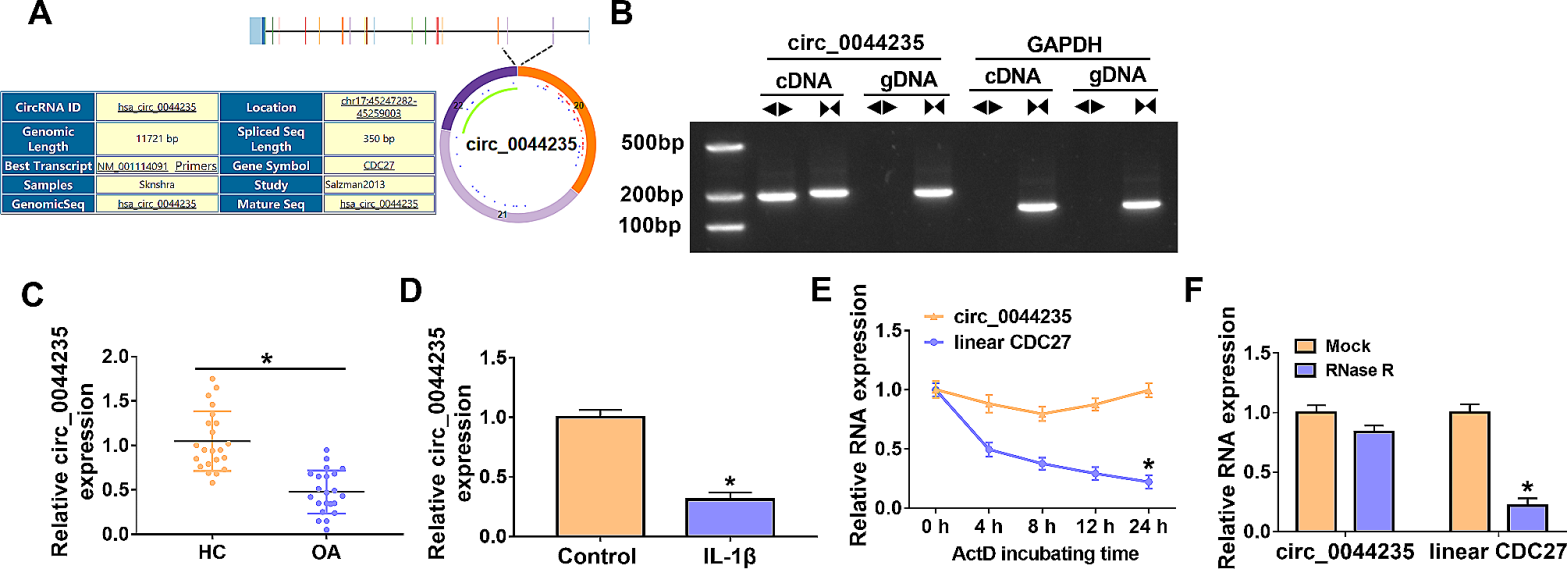
Low level of circ_0044235 in OA cartilage tissues and IL-1β-induced CHON-001 cells. (A) The information of circ_0044235 was shown. (B) DNA gel electrophoresis confirmed the circular structure of circ_0044235. (C and D) Relative circ_0044235 expression in OA cartilage tissues and IL-1β-induced CHON-001 cells was analyzed by qRT-PCR. (E and F) Act D assay or RNase R assay was used to confirm the stability of circ_0044235. *P < 0.05

Circ_0044235 is formed by the back-splicing of exon 20–22 of CDC27 and has a length of 350 bp (Fig. 1A). As shown in Fig. 1B, DNA gel electrophoresis confirmed the circular structure of circ_0044235. QRT-PCR uncovered that circ_0044235 was low expressed in OA cartilage tissues (*n* = 22) (Fig. 1C). QRT-PCR results confirmed that circ_0044235 was down-regulated in IL-1β-treated chondrocytes cells compared with the control group (Fig. 1D). The results of ActD assay and RNase R assay showed that compared to linear CDC27, circ_0044235 expression was stable (Fig. 1E), and it could resist the digestion of RNase R (Fig. 1F).

### Overexpression of circ_0044235 attenuated the effects of IL-1β-mediated CHON-001 cell viability, apoptosis, inflammation, and ECM degradation

**Fig. 2:**
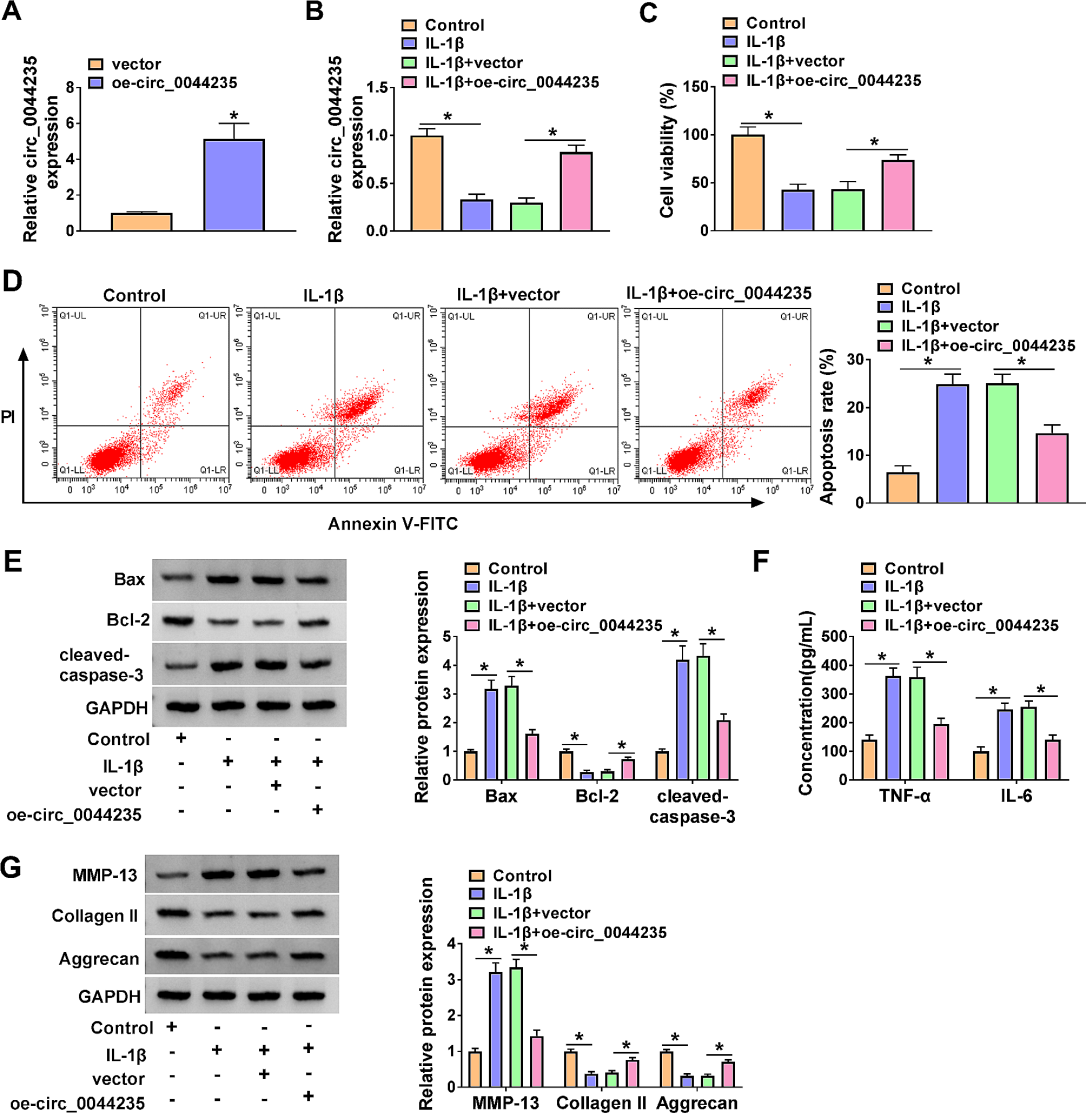
High expression of circ_0044235 reversed IL-1β-induced cell viability, apoptosis, inflammation, and ECM degradation in CHON-001 cells. (A) Relative circ_0044235 expression was determined by qRT-PCR in vector and oe-circ_0044235 group. (B) The expression level of circ_00442235 in IL-1β, IL-1β + vector, or IL-1β + oe-circ_0044235 treated CHON-001 cells or untreated cells (control) was determined by qRT-PCR assay. (C) Cell viability was evaluated by CCK-8 assay. (D) Cell apoptosis was examined by flow cytometry assay. (E) Relative expression of Bax, Bcl-2 and cleaved-caspase-3 was analyzed by western blot. (F) Secretion of inflammatory factor (TNF-α and IL-6) was detected by ELISA assay. (G) The protein expression of MMP-13, Collagen II and Aggrecan was estimated by western blot. *P < 0.05

The transfection efficiency of oe-circ_0044235 was determined by qRT-PCR, and we confirmed that circ_0044235 level was distinctly enhanced after transfection of oe-circ_0044235 (Fig. 2A). As shown in Fig. 2B, transfection of oe-circ_0044235 effectively reversed the decrease of circ_0044235 in CHON-001 cells induced by IL-1β treatment, indicating successful transfection of oe-circ_0044235. CCK-8 assay demonstrated that IL-1β inhibited CHON-001 cell viability, and oe-circ_0044235 could partially attenuate this effect (Fig. 2C). Flow cytometry analysis revealed that IL-1β treatment notably impelled the apoptosis rate of CHON-001 cells, whereas oe-circ_0044235 abolished the impact (Fig. 2D). IL-1β treatment reduced Bcl-2 level and increased Bax and cleaved-caspase-3 levels in CHON-001 cells, while elevation of circ_0044235 attenuated the effects (Fig. 2E). The results of ELISA suggested that IL-1β treatment remarkably facilitated the content of TNF-α and IL-6 in CHON-001 cells, while the effects were recuperated by oe-circ_0044235 (Fig. 2F). Moreover, western blot assay presented that IL-1β treatment increased MMP-13 level and diminished Collagen II and Aggrecan levels in CHON-001 cells, with circ_0044235 overexpression rescued the impacts (Fig. 2G). These data showed that circ_0044235 could alleviate IL-1β-induced CHON-001 cell injury.

### MiR-375 was a target of circ_0044235 in CHON-001 cells

**Fig. 3:**
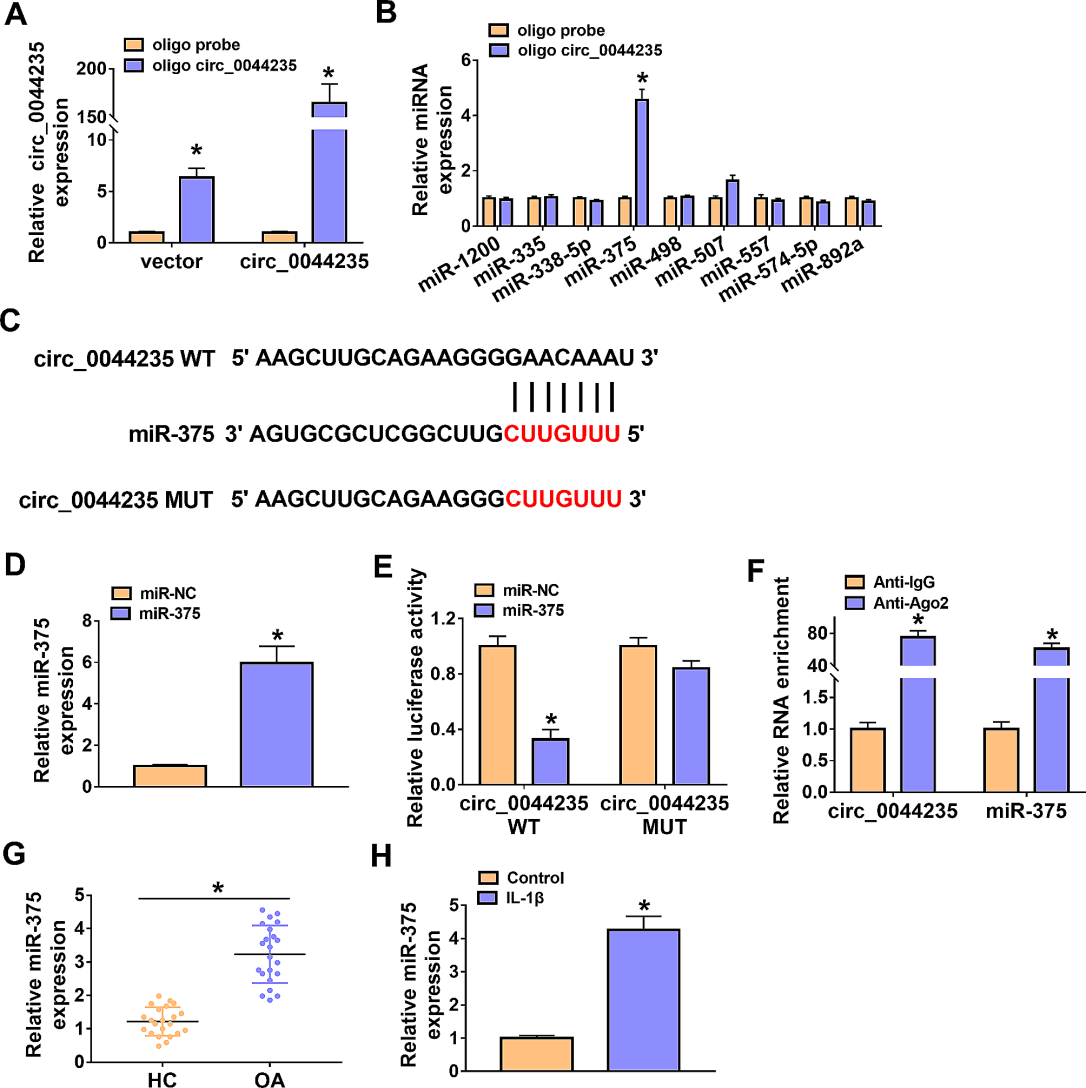
Circ_0044235 directly targeted miR-375. (A) The circ_0044235 level was evaluated by qRT-PCR assay. (B) The expression levels of related miRNAs were detected by qRT-PCR. (C) The predicted binding sites between circ_0044235 and miR-375. (D) Relative miR-375 expression was determined in miR-NC or miR-375 mimic-transfected CHON-001 cells by qRT-PCR. (E) The luciferase activity in CHON-001 cells co-transfected with miR-NC/miR-375 and circ_0044235 WT/circ_0044235 MUT was measured by dual-luciferase reporter assay. (F) The enrichment of circ_0044235 and miR-375 was determined by RIP assay. (G and H) The expression level of miR-375 in OA cartilage tissues and IL-1β-stimulated CHON-001 cells was examined by qRT-PCR assay. *P < 0.05

The circinteractome software (https://circinteractome.irp.nia.nih.gov/) was used to predict the targeted miRNA for circ_0044235, and the top 10 miRNAs were selected as candidate miRNAs. RNA pull-down assay was used to detect the binding ability of circ_0044235 to candidate miRNAs. Biotin-labeled oligo circ_0044235 probe could specifically up-regulate circ_0044235 compared to the control group (Fig. 3A). The results of Fig. 3B showed that only the enrichment of miR-375 was markedly enriched in the oligo circ_0044235 probe (Fig. 3B), so miR-375 was selected as the target of circ_0044235 for our research. The binding sites between miR-375 and circ_0044235 were presented in Fig. 3C. The expression of miR-375 in CHON-001 cells was increased significantly after transfection with miR-375 mimic (Fig. 3D). As presented by dual-luciferase reporter assay, miR-375 apparently impeded the luciferase activity of circ_0044235 WT, and there had no effect on the luciferase activity of circ_0044235 MUT (Fig. 3E). RIP assay implicated that Ago2 drastically enriched circ_0044235 and miR-375 compared with IgG control group (Fig. 3F). Furthermore, miR-375 levels were strikingly elevated in OA cartilage tissues and IL-1β-treated CHON-001 cells (Fig. 3G and H).

### MiR-375 reversed the regulation of circ_0044235 on IL-1β-induced CHON-001 cell injury

**Fig. 4:**
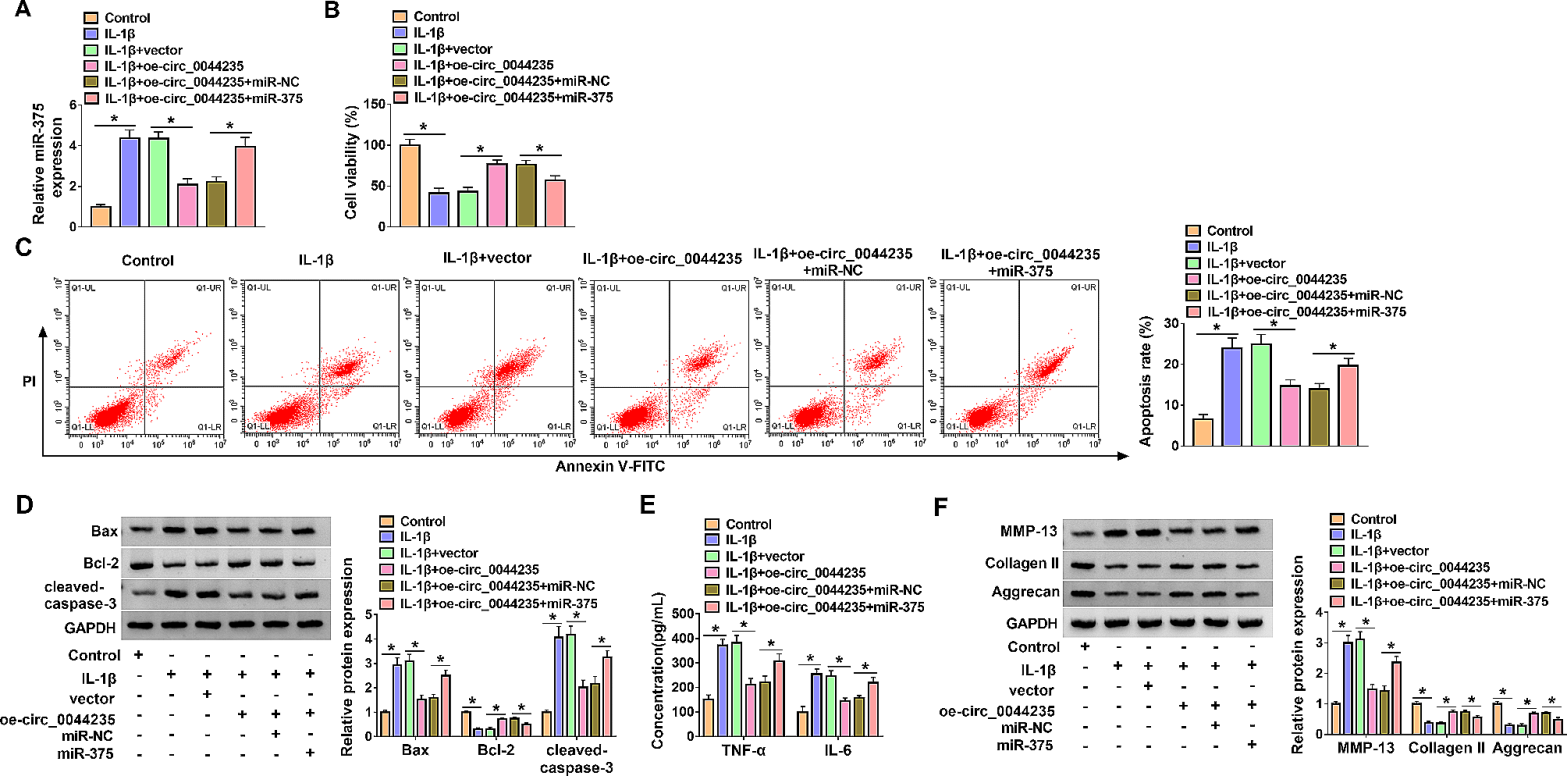
Introduction of miR-375 counteracted the role of circ_0044235 in IL-1β-induced CHON-001 cells. (A) QRT-PCR assay was adopted for miR-375 level in CHON-001 cells. (B and C) The viability and apoptosis of CHON-001 cells were evaluated by CCK-8 assay and flow cytometry analysis, respectively. (D) The protein levels of Bax, Bcl-2 and cleaved-caspase-3 were measured by western blot assay. (E) ELISA was conducted for the levels of TNF-α and IL-6 in CHON-001 cells. (F) Western blot assay was carried out for the protein levels of MMP-13, Collagen II and Aggrecan in CHON-001 cells. *P < 0.05

In IL-1β-induced CHON-001 cells, circ_0044235 overexpression notably inhibited the miR-375 expression, which was rescued by miR-375 mimic (Fig. 4A). CCK-8 and flow cytometry displayed that the promotion effect of circ_0044235 on cell viability and the inhibition effect on cell apoptosis could be eliminated by miR-375 mimic in IL-1β-induced CHON-001 cells (Fig. 4B and C). In IL-1β-stimulated CHON-001 cells, circ_0044235 overexpression up-regulated Bcl-2 protein levels and down-regulated Bax and cleaved-caspase-3 protein levels, while introduction of miR-375 eliminated this effect (Fig. 4D). ELISA consequences manifested that overexpression of circ_0044235 reduced TNF-α and IL-6 concentrations in IL-1β-stimulated CHON-001 cells, while promotion of miR-375 relieved these effects (Fig. 4E). From western blot results, we observed that MMP-13 level was diminished, Collagen II and Aggrecan levels were accelerated in IL-1β-treated CHON-001 cells with circ_0044235 overexpression, while miR-375 overturned the effects (Fig. 4F). Above all, we confirmed that circ_0044235 sponged miR-375 to alleviate IL-1β-induced CHON-001 cell injury.

### Circ_0044235 indirectly regulated PIK3R3 by targeting miR-375

**Fig. 5:**
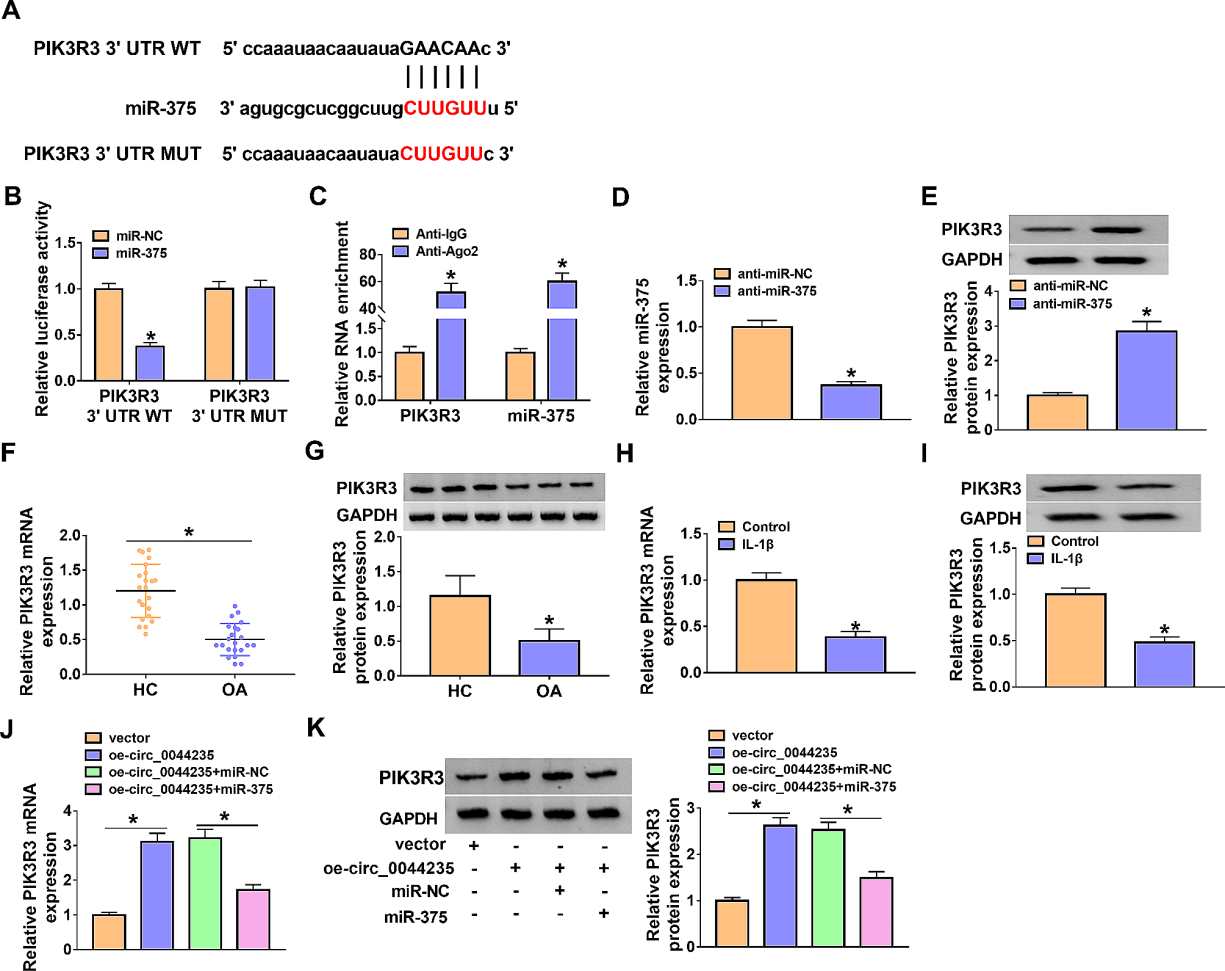
PIK3R3 was a target gene for miR-375. (A) The predicted binding sites between PIK3R3 and miR-375. (B and C) The interaction between miR-375 and PIK3R3 was analyzed by dual-luciferase reporter and RIP assays. (D and E) Relative miR-375 expression and PIK3R3 protein expression in anti-miR-NC or anti-miR-375-transfected CHON-001 cells was analyzed by qRT-PCR and western blot. (F and G) The mRNA and protein levels of PIK3R3 in OA cartilage tissues were determined by qRT-PCR assay and western blot assay, respectively. (H and I) The mRNA and protein levels of PIK3R3 in IL-1β-treated CHON-001 cells was measured by qRT-PCR assay and western blot assay. (J and K) The levels of PIK3R3 mRNA and protein in vector, oe-circ_0044235, oe-circ_0044235 + miR-NC or oe-circ_0044235 + miR-375 treated CHON-001 cells were measured by qRT-PCR assay and western blot assay, respectively. *P < 0.05

StarBase v2.0 software (https://starbase.sysu.edu.cn/)predicted that miR-375 directly targeted PIK3R3 (Fig. 5A). The results of dual-luciferase reporter assay indicated that PIK3R3 3’UTR WT luciferase activity was evidently repressed in CHON-001 cells after transfection with miR-375, while the luciferase activity was not affected in the PIK3R3 3’UTR MUT group (Fig. 5B). The RIP assay results disclosed that PIK3R3 and miR-375 levels were all greatly boosted in Ago2 group in comparison with IgG groups (Fig. 5C). In CHON-001 cells, anti-miR-375 transfection dramatically suppressed miR-375 expression, while PIK3R3 protein level was conspicuously promoted (Fig. 5D and E). As expected, the PIK3R3 mRNA and protein levels were markedly downregulated in OA cartilage tissues compared with the normal group (Fig. 5F and G). Then, we detected the expression of PIK3R3 in IL-1β-treated CHON-001 cells, and found that the mRNA and protein expression of PIK3R3 in IL-1β-treated CHON-001 cells was conspicuously reduced (Fig. 5H and I). In addition, we also observed that overexpression of circ_0044235 increased PIK3R3 expression in chondrocytes, while miR-375 overexpression attenuated this effect (Fig. 5J and K).

### MiR-375 inhibition eliminated IL-1β-induced chondrocyte injury by targeting PIK3R3

**Fig. 6:**
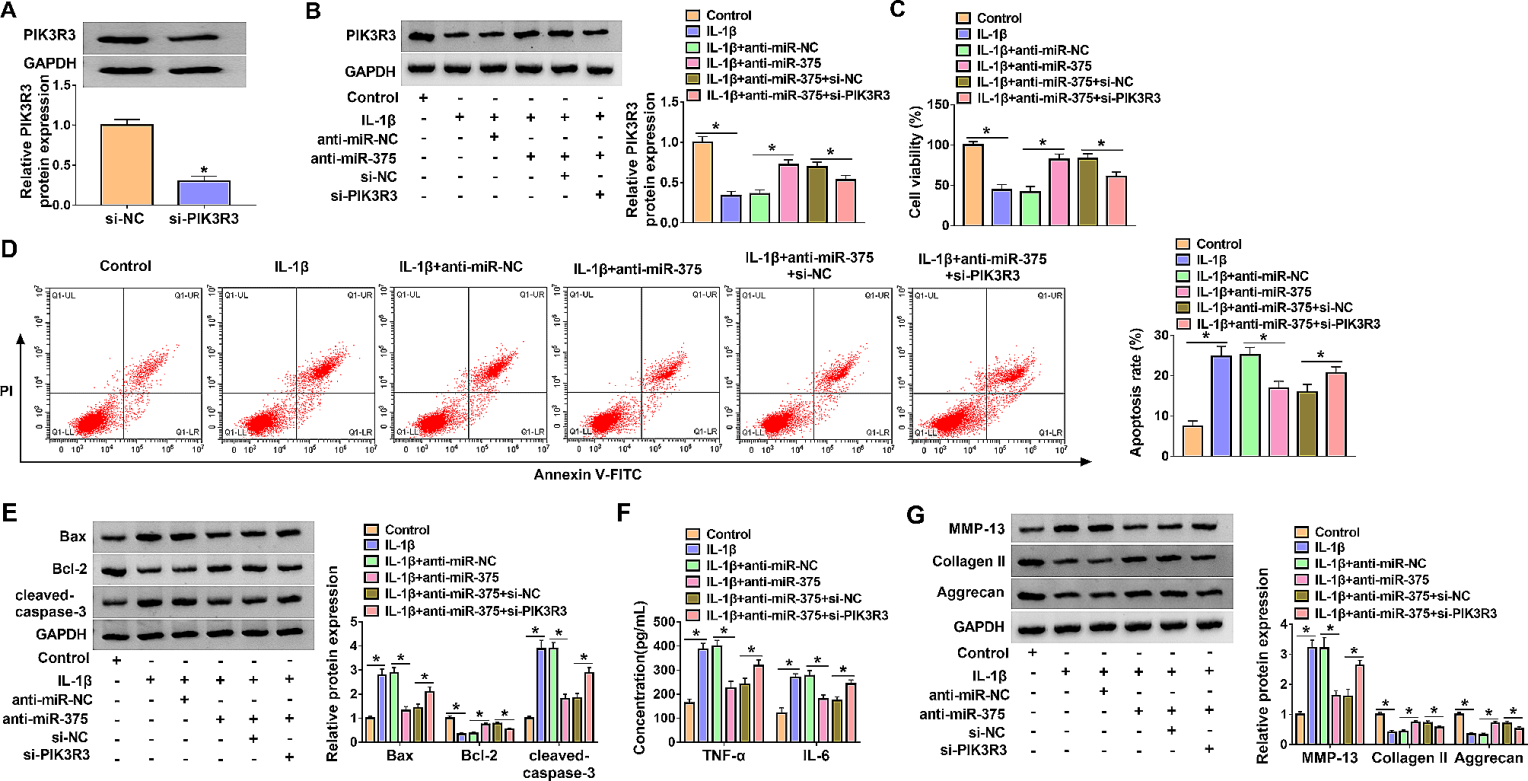
Down-regulation of PIK3R3 partially restored the effect of anti-miR-375 in IL-1β-induced CHON-001 cell injury. (A) The knockdown efficiency of PIK3R3 was measured by western blot. (B) Relative PIK3R3 protein expression in each group was detected by western blot. (C and D) CCK-8 assay and flow cytometry analysis assay were conducted for the viability and apoptosis of CHON-001 cells. (E) The protein levels of Bax, Bcl-2 and cleaved-caspase-3 in CHON-001 cells were measured via western blot assay. (F) ELISA kits were used for the concentrations of TNF-α and IL-6 in CHON-001 cells. (G) Western blot assay was employed for the protein levels of MMP-13, Collagen II and Aggrecan in CHON-001 cells. *P < 0.05

Results shown in Fig. 6A, compared with the control group, si-PIK3R3 prominently reduced the level of PIK3R3, indicating that si-PIK3R3 was successfully transfected (Fig. 6A). As shown in Fig. 6B, miR-375 inhibitor markedly increased PIK3R3 level in IL-1β-treated CHON-001 cells, while si-PIK3R3 abrogated the influence. The results of CCK-8 assay implicated that miR-375 suppression accelerated the viability of CHON-001 cells restricted by IL-1β, with si-PIK3R3 recuperated the impact (Fig. 6C). We found miR-375 silencing restrained cell apoptosis in IL-1β-induced CHON-001 cells, which was abrogated by PIK3R3 knockdown (Fig. 6D). Transfection of anti-miR-375 inhibited the high levels of Bax, cleaved-caspase-3, TNF-α, and IL-6 and the low level of Bcl-2 in IL-1β-induced CHON-001 cells, while si-PIK3R3 relieved the inhibition (Fig. 6E and F). Western blot analysis showed that anti-miR-375 suppressed MMP-13 level and enhanced Collagen II and Aggrecan levels in IL-1β-induced CHON-001 cells, while si-PIK3R3 specially attenuated these effects (Fig. 6G). Above all, we confirmed that miR-375 targeted PIK3R3 to promote IL-1β-induced CHON-001 cell injury.

## Discussion

OA is the result of many factors, and the destruction of articular cartilage caused by the decrease of chondrocytes is one of the important reasons [26]. Besides, inflammatory factors also act a crucial part in OA development. IL-1β is an important inflammatory factor that can induce chondrocyte apoptosis [27]. MMP-13 and Collagen II are closely related to cartilage metabolism. As a matrix metalloproteinase, MMP-13 can effectively degrade Collagen II in chondrocytes, accelerate matrix degradation, and cause the destruction of articular cartilage, leading to the pathogenesis of OA [28, 29]. Therefore, we measured the levels of inflammatory factors and matrix degradation-related protein to assess cell injury under IL-1β induction.

Works have revealed that circRNA participates in the administration of chondrocyte proliferation and inflammatory response, such as circ_0045714, which could accelerate collagen II level chondrocytes proliferation [30]. Down-regulation of ciRS-7 promoted IL-1β-induced chondrocyte apoptosis and inflammatory cytokine release [31]. The results showed that circ_0044235 expression level in OA tissues was decreased. After overexpression of circ_0044235, the IL-1β-induced CHON-001 cell viability was increased, apoptosis rate was reduced, Bcl-2 protein level was augmented, Bax and cleaved caspase-3 levels were diminished, and TNF-α and IL-6 levels were repressed. The consequences exhibited that increasing circ_0044235 could facilitate the proliferation of chondrocytes and inhibit the secretion of inflammatory cytokines induced by IL-1β in chondrocytes.

MiR-375 content was apparently enhanced in IL-1β-induced cartilage cells of OA mouse models, miR-375 inhibition enhanced the ability of chondrocytes to resist oxidative stress and maintain the homeostasis of extracellular matrix metabolism, displaying that miR-375 was an underlying molecular target for OA treatment [17]. Previous research uncovered that miR-375 level was up-regulated in OA cartilage tissues, and the apoptosis rate of chondrocytes was increased when transfection with miR-375 mimic [32]. This work demonstrated that miR-375 level in OA tissues was significantly increased. The rescue experiment showed that elevation of miR-375 weakened the promoting action of circ_0044235 promotion on cell viability, and the decrease of apoptosis rate induced by circ_0044235 up-regulation was also reversed by promotion of miR-375. The effects of circ_0044235 overexpression on Bax, Bcl-2, cleaved caspase-3, and TNF-α and IL-6 were recovered by miR-375 high expression. Circ_0001103 has been reported to improve IL-1β-induced chondrocyte injury through miR-375/SIRT1 axis [18]. This study showed that circ_0044235 could target miR-375, while overexpression of miR-375 relieved the influence of circ_0044235 up-regulation on OA chondrocyte proliferation and inflammatory factor secretion.

PIK3R3 targeted binding to miR-375 was predicted by StarBase v2.0 software. Previous studies have disclosed that PIK3R3 abundance was diminished in OA cells, and miR-1236 could promote the apoptosis of OA chondrocytes by directly inhibiting PIK3R3 [22]. Additionally, *Ran et al.*. demonstrated that circ_0045714/miR-331-3p ameliorated IL-1β-induced HAC cells injury by adjusting PIK3R3 [23]. Similarly, our results were consistent with the above, PIK3R3 level was remarkably reduced in OA. And circ_0044235 could regulate PIK3R3 through miR-375. Recently, siRNAs have been found to regulate tendon homeostasis, osteoporosis and arthritis progression [33–35]. Here, we found that PIK3R3 silencing abolished the role of anti-miR-375 on OA progression.

Of course, our study has some limitations. Due to the limited number of samples, at present we have examined the expression of circ_0044235, miR-375 and PIK3R3 in only 22 samples. To determine the reliability of the study, a larger cohort should be collected for further validation in the future. In addition, we have only conducted studies in human chondrocytes and have not yet carried out in vivo tests. In order to further confirm the possibility of targeting the circ_0044235/miR-375/PIK3R3 axis for the clinical treatment of OA, future in vivo experiments are needed to further verify our conclusion.

**Fig. 7:**
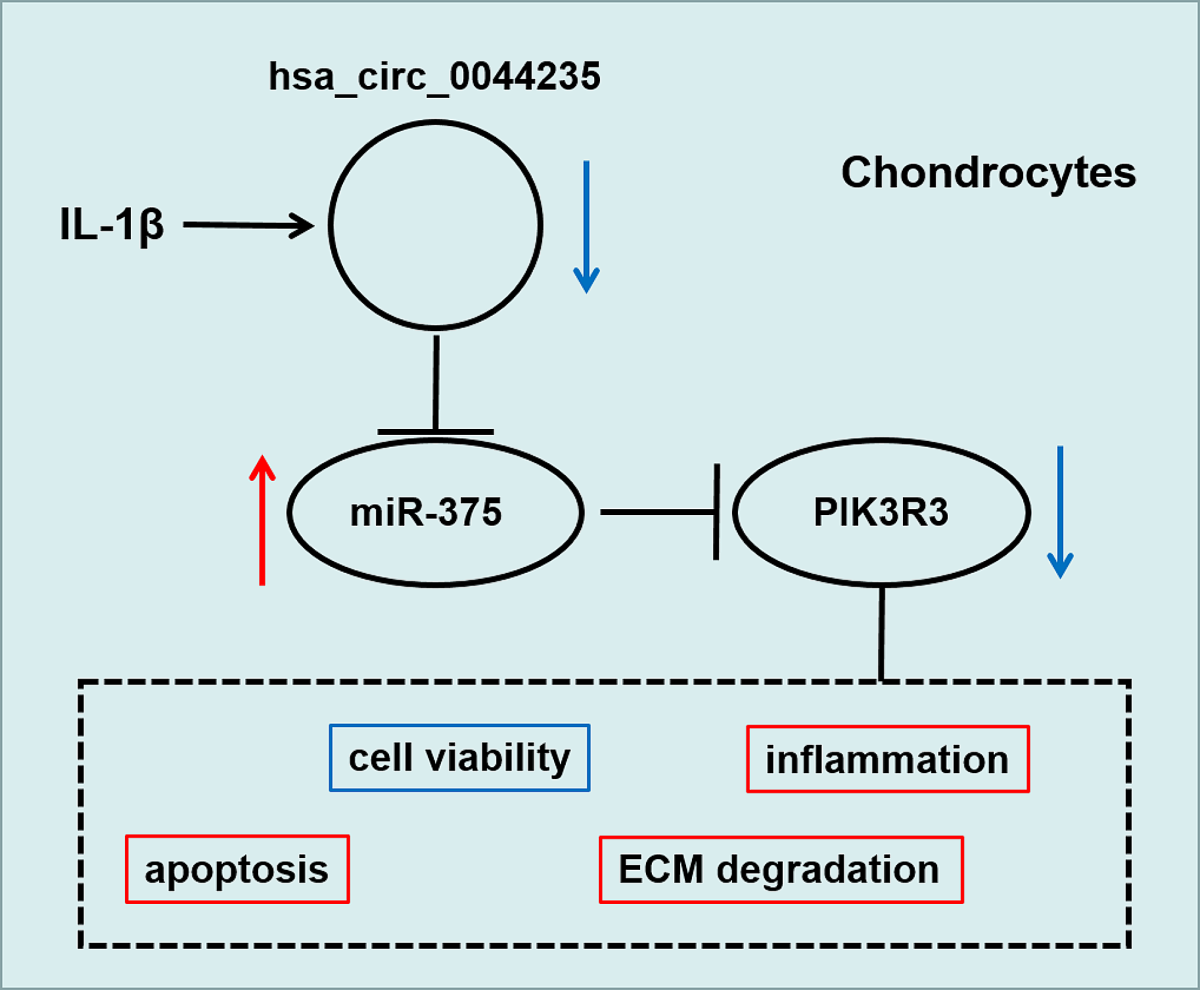
Schematic diagram of this study. Circ_0044235 regulating IL-1β-induced chondrocyte activity, apoptosis, inflammation and ECM degradation

In conclusion, our study reveals a novel regulatory axis that regulates OA progression. Our results showed that circ_0044235 was downregulated in OA patients, and its overexpression alleviated IL-1β-induced chondrocytes apoptosis, inflammation and ECM degradation through the miR-375/PIK3R3 pathway (Fig. 7). The proposed circ_0044235/miR-375/PIK3R3 axis provides a new insight into the formation of OA, and provides a new molecular target for the treatment of OA.

## Data Availability

No datasets were generated or analysed during the current study.
